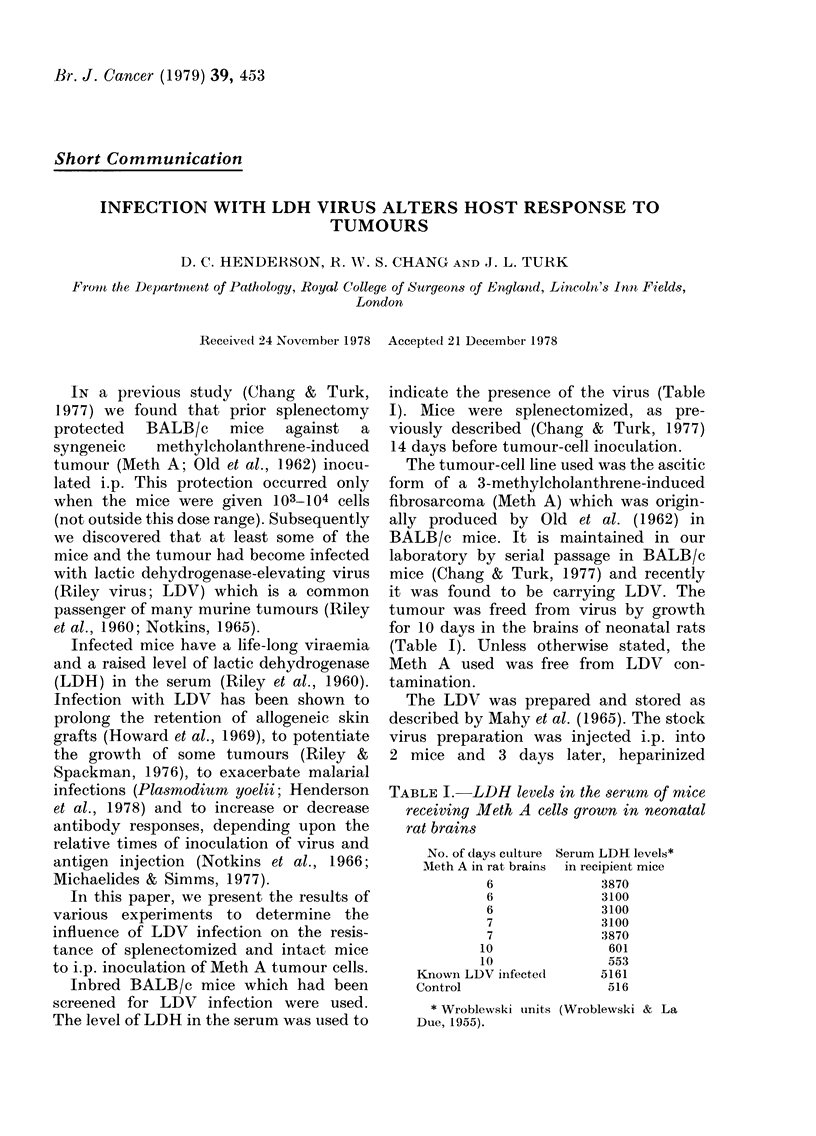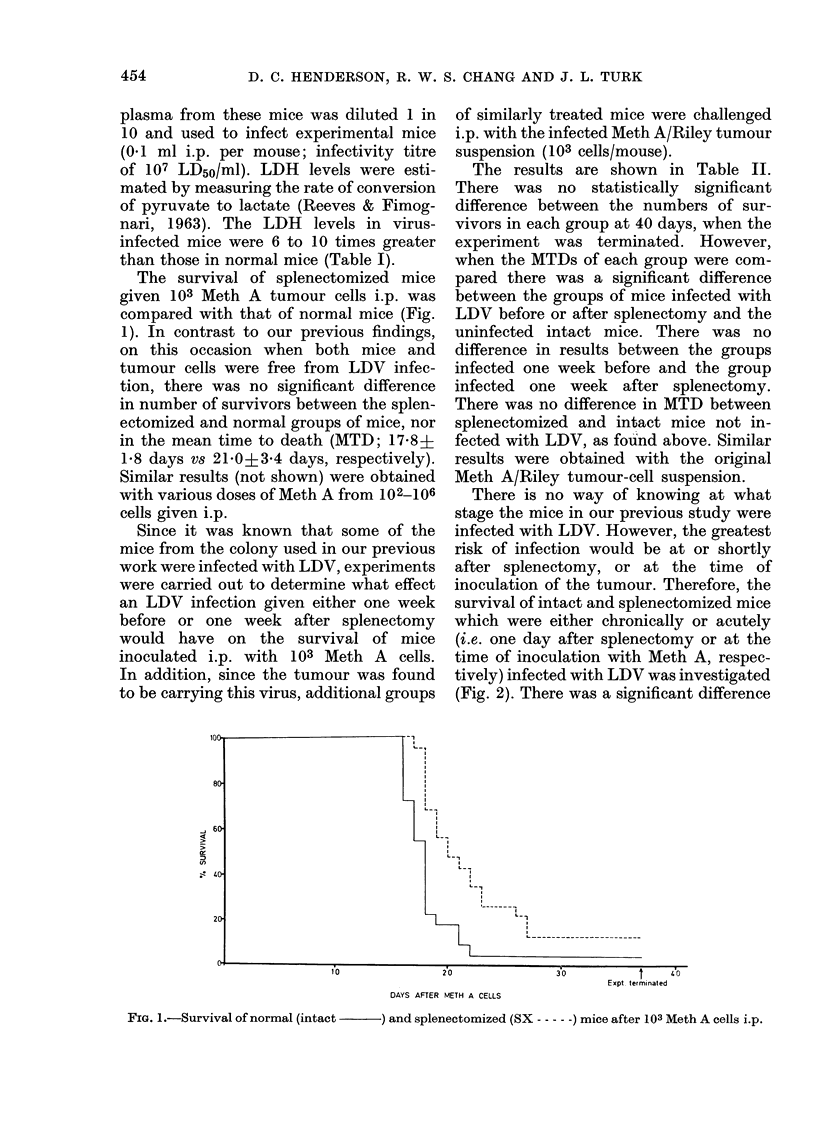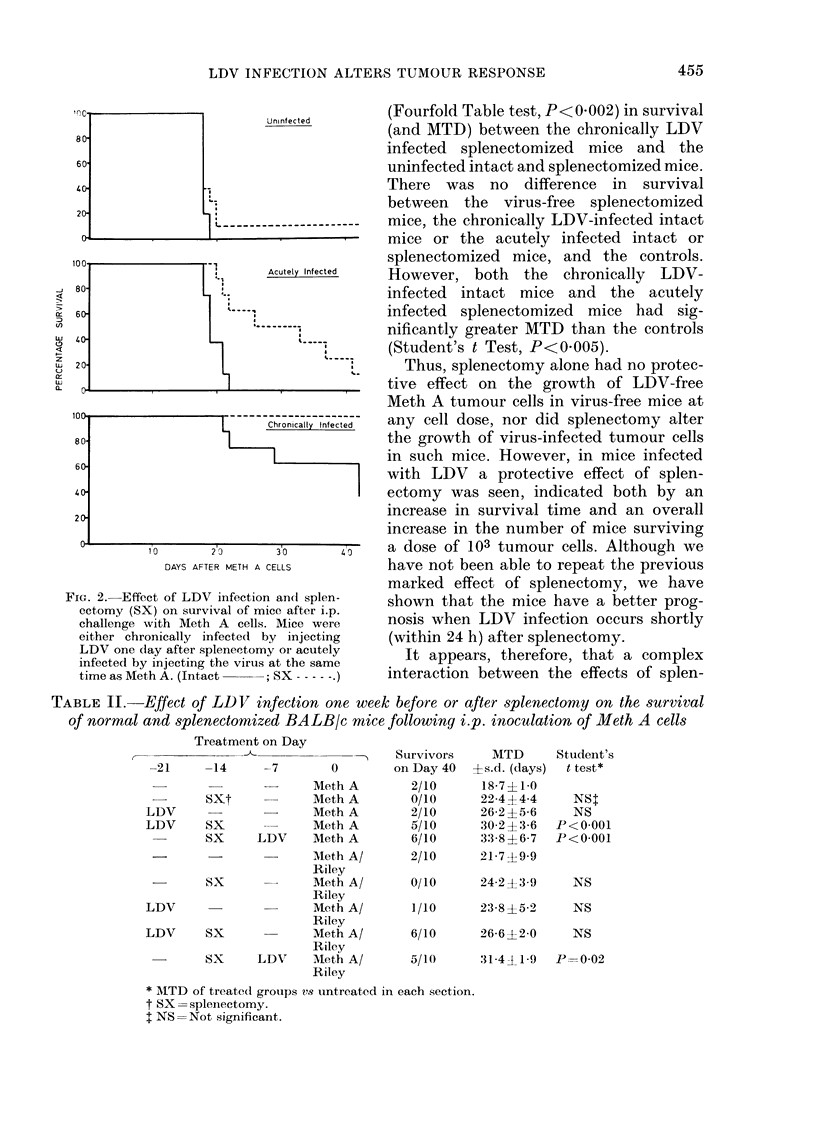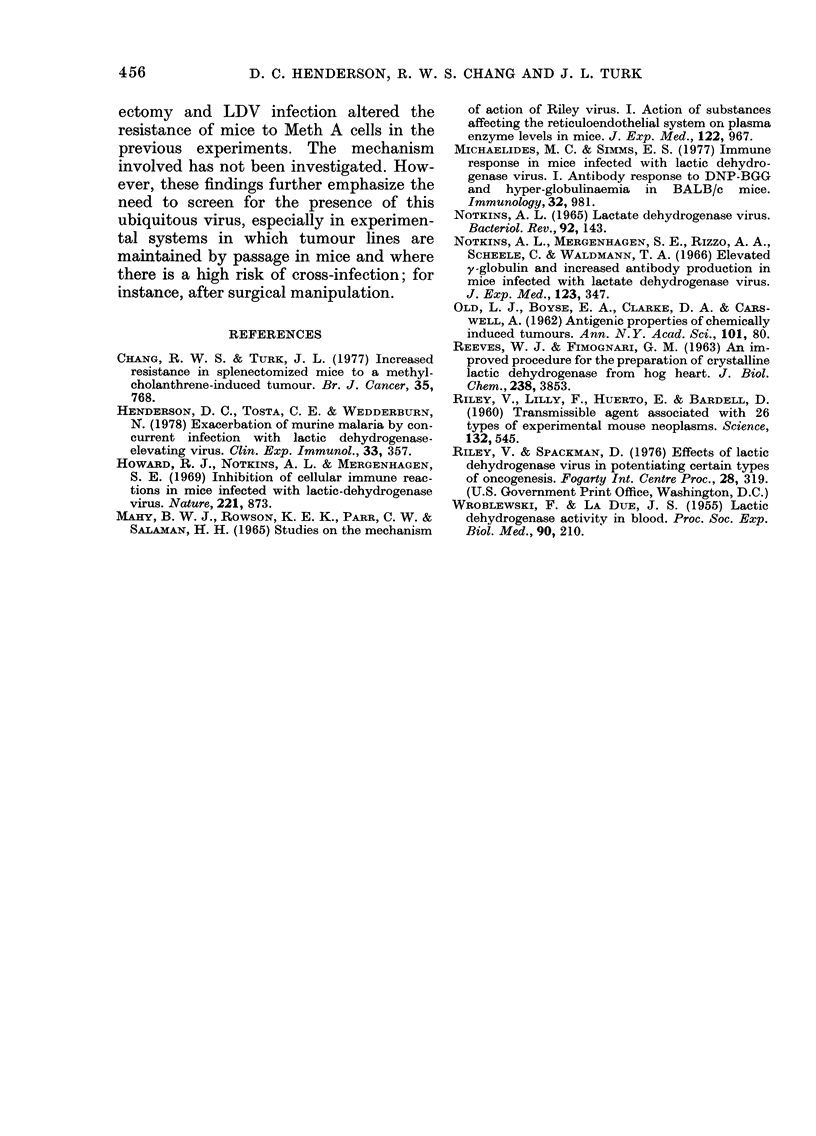# Infection with LDH virus alters host response to tumours.

**DOI:** 10.1038/bjc.1979.81

**Published:** 1979-04

**Authors:** D. C. Henderson, R. W. Chang, J. L. Turk


*Br. J. Cancer* (1979) **39**, 453

***Short Communication***

### INFECTION WITH LDH VIRUS ALTERS HOST RESPONSE TO TUMOURS

D. C. HENDERSON, R. W. S. CHANG AND J. L. TURK

*From the Department of Pathology, Royal College of Surgeons of England, Lincoln's Inn Fields, London*

Received 24 November 1978 Accepted 21 December 1978

IN a previous study (Chang & Turk, 1977) we found that prior splenectomy protected BALB/c mice against a syngeneic methylcholanthrene-induced tumour (Meth A; Old *et al.*, 1962) inoculated i.p. This protection occurred only when the mice were given $10^3$–$10^4$ cells (not outside this dose range). Subsequently we discovered that at least some of the mice and the tumour had become infected with lactic dehydrogenase-elevating virus (Riley virus; LDV) which is a common passenger of many murine tumours (Riley *et al.*, 1960; Notkins, 1965).

Infected mice have a life-long viraemia and a raised level of lactic dehydrogenase (LDH) in the serum (Riley *et al.*, 1960). Infection with LDV has been shown to prolong the retention of allogeneic skin grafts (Howard *et al.*, 1969), to potentiate the growth of some tumours (Riley & Spackman, 1976), to exacerbate malarial infections (*Plasmodium yoelii*; Henderson *et al.*, 1978) and to increase or decrease antibody responses, depending upon the relative times of inoculation of virus and antigen injection (Notkins *et al.*, 1966; Michaelides & Simms, 1977).

In this paper, we present the results of various experiments to determine the influence of LDV infection on the resistance of splenectomized and intact mice to i.p. inoculation of Meth A tumour cells.

Inbred BALB/c mice which had been screened for LDV infection were used. The level of LDH in the serum was used to indicate the presence of the virus (Table I). Mice were splenectomized, as previously described (Chang & Turk, 1977) 14 days before tumour-cell inoculation.

The tumour-cell line used was the ascitic form of a 3-methylcholanthrene-induced fibrosarcoma (Meth A) which was originally produced by Old *et al.* (1962) in BALB/c mice. It is maintained in our laboratory by serial passage in BALB/c mice (Chang & Turk, 1977) and recently it was found to be carrying LDV. The tumour was freed from virus by growth for 10 days in the brains of neonatal rats (Table I). Unless otherwise stated, the Meth A used was free from LDV contamination.

The LDV was prepared and stored as described by Mahy *et al.* (1965). The stock virus preparation was injected i.p. into 2 mice and 3 days later, heparinized

TABLE I.—*LDH levels in the serum of mice receiving Meth A cells grown in neonatal rat brains*

| No. of days culture Meth A in rat brains | Serum LDH levels* in recipient mice |
|---|---|
| 6 | 3870 |
| 6 | 3100 |
| 6 | 3100 |
| 7 | 3100 |
| 7 | 3870 |
| 10 | 601 |
| 10 | 553 |
| Known LDV infected | 5161 |
| Control | 516 |

* Wroblewski units (Wroblewski & La Due, 1955).

plasma from these mice was diluted 1 in 10 and used to infect experimental mice (0·1 ml i.p. per mouse; infectivity titre of $10^7$ $LD_{50}$/ml). LDH levels were estimated by measuring the rate of conversion of pyruvate to lactate (Reeves & Fimognari, 1963). The LDH levels in virus-infected mice were 6 to 10 times greater than those in normal mice (Table I).

The survival of splenectomized mice given $10^3$ Meth A tumour cells i.p. was compared with that of normal mice (Fig. 1). In contrast to our previous findings, on this occasion when both mice and tumour cells were free from LDV infection, there was no significant difference in number of survivors between the splenectomized and normal groups of mice, nor in the mean time to death (MTD; $17{\cdot}8 \pm 1{\cdot}8$ days *vs* $21{\cdot}0 \pm 3{\cdot}4$ days, respectively). Similar results (not shown) were obtained with various doses of Meth A from $10^2$–$10^6$ cells given i.p.

Since it was known that some of the mice from the colony used in our previous work were infected with LDV, experiments were carried out to determine what effect an LDV infection given either one week before or one week after splenectomy would have on the survival of mice inoculated i.p. with $10^3$ Meth A cells. In addition, since the tumour was found to be carrying this virus, additional groups of similarly treated mice were challenged i.p. with the infected Meth A/Riley tumour suspension ($10^3$ cells/mouse).

The results are shown in Table II. There was no statistically significant difference between the numbers of survivors in each group at 40 days, when the experiment was terminated. However, when the MTDs of each group were compared there was a significant difference between the groups of mice infected with LDV before or after splenectomy and the uninfected intact mice. There was no difference in results between the groups infected one week before and the group infected one week after splenectomy. There was no difference in MTD between splenectomized and intact mice not infected with LDV, as found above. Similar results were obtained with the original Meth A/Riley tumour-cell suspension.

There is no way of knowing at what stage the mice in our previous study were infected with LDV. However, the greatest risk of infection would be at or shortly after splenectomy, or at the time of inoculation of the tumour. Therefore, the survival of intact and splenectomized mice which were either chronically or acutely (*i.e.* one day after splenectomy or at the time of inoculation with Meth A, respectively) infected with LDV was investigated (Fig. 2). There was a significant difference

Fig. 1.—Survival of normal (intact ———) and splenectomized (SX - - - - -) mice after $10^3$ Meth A cells i.p.

FIG. 2.—Effect of LDV infection and splenectomy (SX) on survival of mice after i.p. challenge with Meth A cells. Mice were either chronically infected by injecting LDV one day after splenectomy or acutely infected by injecting the virus at the same time as Meth A. (Intact ———; SX - - - - -.)

(Fourfold Table test, $P<0\cdot002$) in survival (and MTD) between the chronically LDV infected splenectomized mice and the uninfected intact and splenectomized mice. There was no difference in survival between the virus-free splenectomized mice, the chronically LDV-infected intact mice or the acutely infected intact or splenectomized mice, and the controls. However, both the chronically LDV-infected intact mice and the acutely infected splenectomized mice had significantly greater MTD than the controls (Student's $t$ Test, $P<0\cdot005$).

Thus, splenectomy alone had no protective effect on the growth of LDV-free Meth A tumour cells in virus-free mice at any cell dose, nor did splenectomy alter the growth of virus-infected tumour cells in such mice. However, in mice infected with LDV a protective effect of splenectomy was seen, indicated both by an increase in survival time and an overall increase in the number of mice surviving a dose of $10^3$ tumour cells. Although we have not been able to repeat the previous marked effect of splenectomy, we have shown that the mice have a better prognosis when LDV infection occurs shortly (within 24 h) after splenectomy.

It appears, therefore, that a complex interaction between the effects of splen-

TABLE II.—*Effect of LDV infection one week before or after splenectomy on the survival of normal and splenectomized BALB/c mice following i.p. inoculation of Meth A cells*

| Treatment on Day | | | | Survivors | MTD | Student's |
|---|---|---|---|---|---|---|
| -21 | -14 | -7 | 0 | on Day 40 | ±s.d. (days) | $t$ test* |
| — | — | — | Meth A | 2/10 | 18·7±1·0 | |
| — | SX† | — | Meth A | 0/10 | 22·4±4·4 | NS‡ |
| LDV | — | — | Meth A | 2/10 | 26·2±5·6 | NS |
| LDV | SX | — | Meth A | 5/10 | 30·2±3·6 | $P<0\cdot001$ |
| — | SX | LDV | Meth A | 6/10 | 33·8±6·7 | $P<0\cdot001$ |
| — | — | — | Meth A/ Riley | 2/10 | 21·7±9·9 | |
| — | SX | — | Meth A/ Riley | 0/10 | 24·2±3·9 | NS |
| LDV | — | — | Meth A/ Riley | 1/10 | 23·8±5·2 | NS |
| LDV | SX | — | Meth A/ Riley | 6/10 | 26·6±2·0 | NS |
| — | SX | LDV | Meth A/ Riley | 5/10 | 31·4±1·9 | $P=0\cdot02$ |

* MTD of treated groups *vs* untreated in each section.

† SX = splenectomy.

‡ NS = Not significant.

ectomy and LDV infection altered the resistance of mice to Meth A cells in the previous experiments. The mechanism involved has not been investigated. However, these findings further emphasize the need to screen for the presence of this ubiquitous virus, especially in experimental systems in which tumour lines are maintained by passage in mice and where there is a high risk of cross-infection; for instance, after surgical manipulation.

### REFERENCES

CHANG, R. W. S. & TURK, J. L. (1977) Increased resistance in splenectomized mice to a methylcholanthrene-induced tumour. *Br. J. Cancer*, **35**, 768.

HENDERSON, D. C., TOSTA, C. E. & WEDDERBURN, N. (1978) Exacerbation of murine malaria by concurrent infection with lactic dehydrogenase-elevating virus. *Clin. Exp. Immunol.*, **33**, 357.

HOWARD, R. J., NOTKINS, A. L. & MERGENHAGEN, S. E. (1969) Inhibition of cellular immune reactions in mice infected with lactic-dehydrogenase virus. *Nature*, **221**, 873.

MAHY, B. W. J., ROWSON, K. E. K., PARR, C. W. & SALAMAN, H. H. (1965) Studies on the mechanism of action of Riley virus. I. Action of substances affecting the reticuloendothelial system on plasma enzyme levels in mice. *J. Exp. Med.*, **122**, 967.

MICHAELIDES, M. C. & SIMMS, E. S. (1977) Immune response in mice infected with lactic dehydrogenase virus. I. Antibody response to DNP-BGG and hyper-globulinaemia in BALB/c mice. *Immunology*, **32**, 981.

NOTKINS, A. L. (1965) Lactate dehydrogenase virus. *Bacteriol. Rev.*, **92**, 143.

NOTKINS, A. L., MERGENHAGEN, S. E., RIZZO, A. A., SCHEELE, C. & WALDMANN, T. A. (1966) Elevated $\gamma$-globulin and increased antibody production in mice infected with lactate dehydrogenase virus. *J. Exp. Med.*, **123**, 347.

OLD, L. J., BOYSE, E. A., CLARKE, D. A. & CARSWELL, A. (1962) Antigenic properties of chemically induced tumours. *Ann. N.Y. Acad. Sci.*, **101**, 80.

REEVES, W. J. & FIMOGNARI, G. M. (1963) An improved procedure for the preparation of crystalline lactic dehydrogenase from hog heart. *J. Biol. Chem.*, **238**, 3853.

RILEY, V., LILLY, F., HUERTO, E. & BARDELL, D. (1960) Transmissible agent associated with 26 types of experimental mouse neoplasms. *Science*, **132**, 545.

RILEY, V. & SPACKMAN, D. (1976) Effects of lactic dehydrogenase virus in potentiating certain types of oncogenesis. *Fogarty Int. Centre Proc.*, **28**, 319. (U.S. Government Print Office, Washington, D.C.)

WROBLEWSKI, F. & LA DUE, J. S. (1955) Lactic dehydrogenase activity in blood. *Proc. Soc. Exp. Biol. Med.*, **90**, 210.